# From Mice to Mole-Rats: Species-Specific Modulation of Adult Hippocampal Neurogenesis

**DOI:** 10.3389/fnins.2017.00602

**Published:** 2017-10-30

**Authors:** Maria K. Oosthuizen

**Affiliations:** Department of Zoology and Entomology, University of Pretoria, Pretoria, South Africa

**Keywords:** African mole-rats, breeding, laboratory rodents, neurogenesis, non-breeding, social, solitary

## Abstract

Rodent populations living in their natural environments have very diverse ecological and life history profiles that may differ substantially from that of conventional laboratory rodents. Free-living rodents show species-specific neurogenesis that are dependent on their unique biology and ecology. This perspective aims to illustrate the benefit of studying wild rodent species in conjunction with laboratory rodents. African mole-rats are discussed in terms of habitat complexity, social structures, and longevity. African mole-rats are a group of subterranean rodents, endemic to Africa, that show major differences in both intrinsic and extrinsic traits compared to the classical rodent models. Mole-rats exhibit a spectrum of sociality within a single family, ranging from solitary to eusocial. This continuum of sociality provides a platform for comparative testing of hypotheses. Indeed, species differences are apparent both in learning ability and hippocampal neurogenesis. In addition, social mole-rat species display a reproductive division of labor that also results in differential hippocampal neurogenesis, independent of age, offering further scope for comparison. In conclusion, it is evident that neurogenesis studies on conventional laboratory rodents are not necessarily representative, specifically because of a lack of diversity in life histories, uniform habitats, and low genetic variability. The observed level of adult neurogenesis in the dentate gyrus is the result of an intricate balance between many contributing factors, which appear to be specific to distinct groups of animals. The ultimate understanding of the functional and adaptive role of adult neurogenesis will involve research on both laboratory animals and natural rodent populations.

## Introduction

It is widely accepted that adult neurogenesis is restricted to two neurogenic regions in the mammalian brain, the subventricular zone of the lateral ventricles and the subgranular zone of the hippocampus (Gage, 2000). Adult hippocampal neurogenesis (AHN) is a dynamic process that has been implicated in hippocampus dependent cognitive functions and both positive and negative regulators of AHN have been described (Aimone et al., 2014). However, the majority of our knowledge originates from studies performed on a few laboratory species that are highly inbred and are maintained in stable laboratory conditions (Kempermann, 2012). Intrinsic and extrinsic traits of wild rodents that were not raised in the laboratory, may differ significantly from that of conventional laboratory animals in factors such as genetic variability, social structure, habitat complexity, and longevity, all of which can influence AHN (Kuhn et al., 1996; Kempermann et al., 1997a; Kozorovitskiy and Gould, 2004). Hence, the main drivers of AHN may interact in diverse and unpredictable ways to produce opposite patterns of AHN in wild rodents compared to conventional laboratory rodents, or the drivers may be altogether different.

Since laboratory rodents are frequently used as models for disease-related medical research, it is imperative to understand their limitations and appreciate that life history may influence experimental results. Although the functional significance of AHN may overlap in diverse taxonomic groups, the adaptive value thereof may differ considerably across taxa. Indeed, substantial differences in the extent and magnitude of neurogenesis between mice and humans have been revealed (Jessberger and Gage, 2014). Hence, the investigation of species other than conventional laboratory animals with different traits may provide a useful comparative framework to investigate the adaptive advantage of AHN.

**Figure 1:**
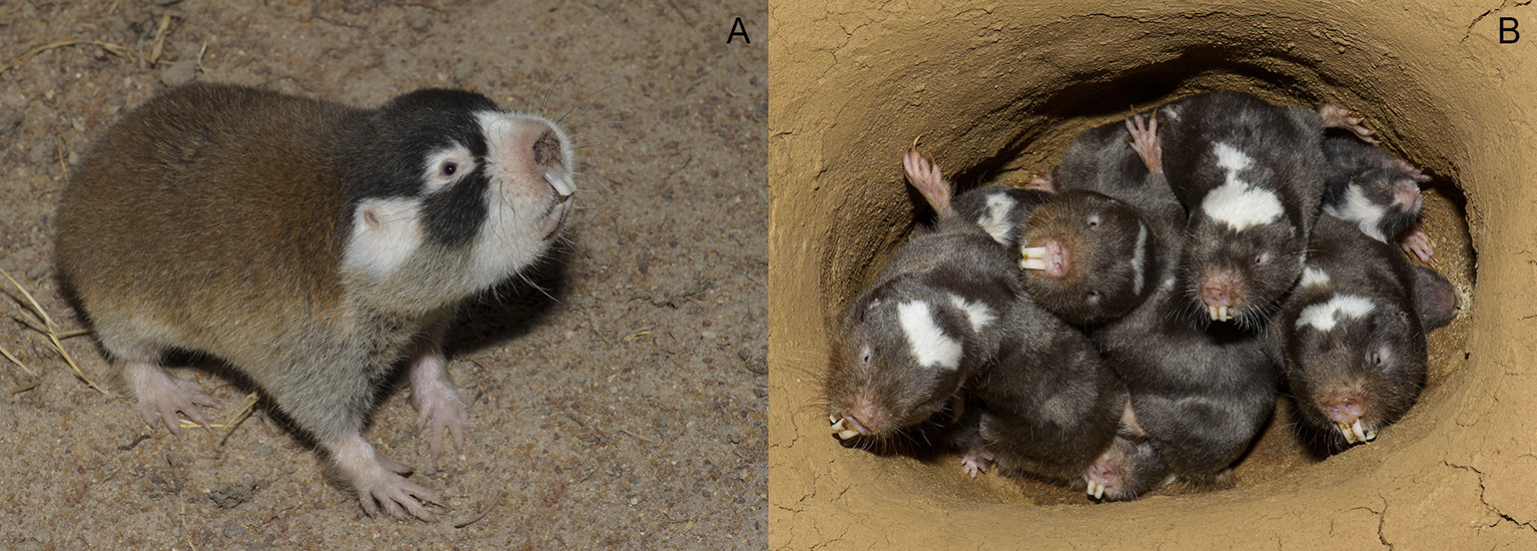
**(A)** Solitary Cape mole-rat. **(B)** A colony of social Damaraland mole-rats.

The aim of this work is to highlight that both intrinsic and extrinsic traits of non-conventional rodent species can deviate significantly from that of conventional laboratory animals, and may affect the modulation of AHN very differently depending on species specific requirements. A relevant example is the African mole-rats, a group of subterranean rodents, endemic to Africa. African mole-rats are rodent moles that belong to the family Bathyergidae, which differ radically from laboratory rodents in a number of contexts such as environmental niche, social structures, behavior, and longevity (Figure 1). These factors will be compared between laboratory rodents and mole-rats to provide a perspective on differences in the adaptive value of neurogenesis in the two groups of rodents.

## Neurogenesis in conventional laboratory rodent models

Adult hippocampal neurogenesis (AHN) has been extensively studied in laboratory rodents, both in the context of basal and experimentally manipulated levels of AHN. Both positive and negative regulators of neurogenesis have been identified, some factors are context dependent and may serve as positive, and negative regulators. The factors mentioned below is by no means a complete survey of all potential regulators, merely ones deemed relevant for the ensuing discussion.

### Habitat complexity

Laboratory animals frequently live in a relatively constant habitat, lacking many of the external factors that can influence their biology. Several studies have demonstrated increased AHN in laboratory rodents in response to enriched environments (Kempermann et al., 1997b, 1998; Nillson et al., 1999; Brown et al., 2003). Habitat complexity increases the need for behavioral flexibility (Amrein, 2015), thus free-living rodent species living in very complex habitats have been shown to exhibit much higher neurogenesis compared to animals that inhabit less complex habitats (Amrein et al., 2007; Garthe et al., 2009; Cavegn et al., 2013).

### Social environment

The social environment of laboratory animals can exert both positive and negative effects on neurogenesis, depending on the circumstances. Laboratory rodents such as mice and rats are typically communal species and social interactions have been shown to significantly affect the regulation of adult neurogenesis in the hippocampus (Fowler et al., 2008; Lieberwirth and Wang, 2012). Social status can influence the rate of neurogenesis where, in laboratory rodents, animals with a higher status typically show more neurogenesis than the ones with a lower social status (Gould et al., 1997, 1998; Kozorovitskiy and Gould, 2004; Thomas et al., 2007; Wu et al., 2014). Variation in estrogen levels may be the underlying mechanism responsible for differences in AHN between dominant and subordinate animals. Dominant animals typically have a higher probability of breeding, and breeding individuals usually exhibit higher levels of estrogen. Estrogen has been shown to play a role in proliferation, the survival as well as the activation of the new neurons (Fowler et al., 2008). Following ovariectomy, AHN was reduced but this could be reversed by estrogen replacement (Tanapat et al., 1999). Cell proliferation in the dentate gyrus (DG) of the hippocampus of laboratory rat females also fluctuates according to the estrous cycle, with higher cell proliferation when more estrogen is present (Tanapat et al., 1999).

Stress hormones can have positive or negative effects on AHN, depending on the type of stressor and whether the stress is acute or chronic (Schoenfeld and Gould, 2012). High levels of corticosterone in response to social isolation causes a reduction in neurogenesis and also decreases performance in other behavioral tests (Stranahan et al., 2006). This effect seems to be larger in females compared to males (Westenbroek et al., 2004). In contrast, exercise (running) induces lower levels of corticosterone and is associated with an increase in AHN (Stranahan et al., 2006).

### Age

Age is commonly viewed as a potent negative regulator of AHN since there is a dramatic decline in neurogenesis that appears to be age related in most mammals investigated, laboratory rodents included (Kuhn et al., 1996; Amrein et al., 2004; Ben Abdallah et al., 2010). This downregulation of neurogenesis is not correlated with the environment or other species-specific traits such as longevity or developmental strategy (Amrein et al., 2004). Technically, this decrease occurs relatively early in life, after which the level of neurogenesis remains relatively stable.

### Cognitive activity

A vast number of studies suggest a link between learning, memory, and adult neurogenesis. Initial studies showed that learning increases hippocampal neurogenesis (Gould et al., 1999a,b) which accordingly enhances spatial memory (Snyder et al., 2005; Winocur et al., 2006). In turn, learning impairments are associated with a reduction in hippocampal neurogenesis (Lemaire et al., 2000). However, more recent evidence suggests that this is highly species and context dependent and the results are not always consistent (Dobrossy et al., 2003; Jaholkowski et al., 2009; Groves et al., 2013; Duarte-Guterman et al., 2015). In some cases, neurogenesis has no effect on spatial memory at all (Groves et al., 2013). The mixed results may be attributed to the type of behavioral tests performed or the history and age of the animals, or a combination of factors.

## Comparing laboratory models with the mole-rat model

Some factors that can influence AHN in rodents exhibit parallel features in laboratory rodent models and the mole-rat model, with the difference that it occurs naturally in mole-rats but are human induced in laboratory rodents. Other factors are distinctly different for the two models.

### Habitat complexity

Habitat complexity, or the lack thereof, can influence AHN in rodents depending on the need for behavioral flexibility (Amrein, 2015). Laboratory rodents have probably adapted to their relatively uniform and confined laboratory habitat over many generations in captivity, with accompanying alterations in behavioral needs (Toth et al., 2011). Although in a different spatial context, the sealed burrow systems of mole-rats is devoid of light and lack many other sensory cues available to aboveground living rodents (Burda et al., 1990). Mole-rats thus present a model that naturally inhabits a uniform environment. In addition, even the simplistic environment of mole-rats shows interspecific variation in terms of length and complexity of the tunnels, depending on the social structure of the species, presenting opportunity for comparisons.

### Social structure

Laboratory mice and rats are generally classified as social and polygamous but the social structure is not rigidly fixed (Lund, 1975; Hedrich, 2012). As a taxonomic group, mole-rats show much more diverse and complex social organizations compared to laboratory animals. Bathyergids exhibit a spectrum of sociality within a single taxonomic family, ranging from strictly solitary to highly social species (Faulkes et al., 1997). Solitary species are typically polygamous whereas social species tend to be more monogamous. Social mole-rats live in family groups that exhibit a distinct reproductive division of labor (Jarvis, 1981; Bennett, 1988). Reproduction is restricted to a single female and one or two males, while the remainder of the colony comprises overlapping generations of subordinate animals that are reproductively suppressed. Mole-rats breed cooperatively, and the non-breeding individuals assist with the rearing of offspring and maintenance of the tunnel system (Jarvis, 1981; Bennett, 1988). Social mole-rat colonies exhibit linear dominance hierarchies where larger animals are dominant over smaller animals (Jacobs et al., 1991), but breeding animals are always dominant over non-breeding animals. In other rodents, both dominance and reproductive status have been shown to influence AHN (Tanapat et al., 1999; Kozorovitskiy and Gould, 2004). Therefore, the difference in social structures between species and the within species status differences in the mole-rat model provide abundant opportunities for empirical testing of predictions in a comparative setting.

### Age and longevity

Laboratory mice and rats have maximum lifespans of under 5 years (Gorbunova et al., 2008), whereas their free-living counterparts may have a much shorter life expectancy. Similar sized mole-rats, especially the social species, can attain ages three to six-fold that of laboratory animals. In captivity, the age of 16 years have been recorded for social *Fukomys* mole-rats (Dammann et al., 2011) and the age of 32 years for naked mole-rats (*Heterocephalus glaber*) (Buffenstein and Jarvis, 2002). An exponential decline in AHN is apparent in both long and short-lived species, but the slower maturation of longer lived species may offer a larger window for experimental manipulation of the baseline AHN.

## Mole-rat neurogenesis

Morphologically, the dentate gyrus of mole-rats is comparatively smaller than that of other rodents, with fewer granule cells (Amrein et al., 2014). Mole-rats in general have very low levels of neurogenesis in the hippocampus (Amrein et al., 2014; Penz et al., 2015; Oosthuizen and Amrein, 2016). Normalized proliferating cell numbers of mole-rats are comparable with that of other rodents however the normalized young neurons are lower (Amrein et al., 2014).

### Habitat complexity

The lower survival rate of young neurons in mole-rats supports a habitat-dependent modulation of neurogenesis. The habitat complexity of mole-rat burrow systems is very low when compared to the highly complex three-dimensional environments of surface dwelling rodents. The sealed burrow systems of mole-rats lack external cues and therefore present a very homogenous and stable habitat (Burda et al., 1990).

Despite the overall low habitat complexity, the length and complexity of mole-rat burrows differ between species, it depends on a number of factors including sociality, habitat type, resource availability, and population density (Le Comber et al., 2002). The burrow systems of Damaraland mole-rats (*Fukomys damarensis*) can reach up to 2 km in length (Bennett and Faulkes, 2000), while burrow systems of solitary species such as the Cape mole-rat (*Georychus capensis*) are generally much shorter (Thomas et al., 2012). Within the context of the subterranean niche, interspecies differences in hippocampal neurogenesis that is consistent with the relative length and complexity of the burrows of the different species, is evident. The social and solitary species have similar numbers of granule cells despite size differences in the species [Cape: 150–200 g; Highveld (*Cryptomys hottentotus pretoriae*; intermediately social species): 80–120 g; Damaraland: 120–150 g; Bennett and Faulkes, 2000, pers. obs.], however both the Highveld and Damaraland mole-rats have more proliferating cells compared to Cape mole-rats (Ki67 staining) (Amrein et al., 2014; Oosthuizen and Amrein, 2016). The numbers of young neurons show large within species variation, thus young neuron numbers of the individual species show some overlap.

### Social status

**Figure 2:**
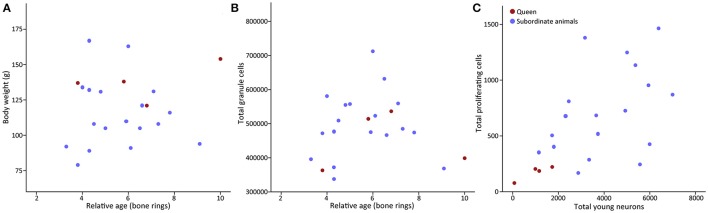
Scatter plots of relative age x body weight, total granule cells × relative age, and proliferation × young neurons in Damaraland mole-rats. **(A)** The body weight of Damaraland mole-rats does not increase with relative age, **(B)** total granule cells remains stable with relative age, and **(C)** a scatter plot of proliferating cells (Ki67) and young neurons (PSA-NCAM) in dominant and subordinate Damaraland mole-rats (Modified from (Oosthuizen and Amrein, 2016), with permission from Elsevier).

Despite the low rate of neurogenesis, a status dependent amount of hippocampal neurogenesis is evident in the social Damaraland mole-rats. Seemingly in contrast with results from laboratory rodents, the breeding females, or queens, have lower numbers of both proliferating cells and young neurons compared to subordinate colony members (Oosthuizen and Amrein, 2016) (Figure 2). A similar occurrence is observed in the naked mole-rat, where breeding animals were found to have significantly less young neurons, as visualized by doublecortin (DCX) immunoreactive neurons, compared to the non-breeding animals (Peragine et al., 2014). Differential AHN in breeding and non-breeding mole-rats may potentially have an endocrinological basis. Both reproductive hormones and stress hormones have been shown to modulate neurogenesis in laboratory rodents (Cameron and Gould, 1994; Gould and Tanapat, 1999; Tanapat et al., 1999).

In highly social species such as the Damaraland mole-rat and the naked mole-rat, the breeding females have higher estrogen levels compared to non-breeding animals (Bennett and Jarvis, 1988; Faulkes et al., 1990; Bennett, 1994), thus one would also expect an upregulation of neurogenic cells, yet the opposite is true (Peragine et al., 2014; Oosthuizen and Amrein, 2016). In the case of mole-rats, estrogen appears to rather downregulate AHN. Similarly, cell proliferation is inhibited in the dentate gyrus of female meadow voles in the breeding season, when high estrogen levels are present, compared to females out of the breeding season, although more cells survive in the reproductively active females (Galea and McEwen, 1999; Ormerod and Galea, 2001). A potential mechanism for the disparity in the effect of estrogen on AHN may be related to the density of the estrogen receptor α in the dentate gyrus. In voles, estrogen increased the density of the estrogen receptor α (ERα) (Fowler et al., 2005), however this increase was region specific and no difference was observed in the dentate gyrus. In mole-rats, non-breeding Damaraland mole-rat females express lower levels of ERα compared to the breeders in brain regions important for reproduction (Voigt et al., 2014). The density of the ERα has not been investigated in the DG of mole-rats thus far, but could potentially also show differential expression between breeder and non-breeder mole-rats. In naked mole-rats, no significant relationships could be identified between circulating gonadal steroids and DCX (Peragine et al., 2014), however this do not exclude potential differences in proliferating cells or receptors for the hormones.

Stress hormones have not been exhaustively investigated in mole-rats in terms of social status in the colony. It appears that when the social structure is stable, there is no difference cortisol levels between breeding and non-breeding animals (Clarke and Faulkes, 1998; Clarke et al., 2001), but instability in the social structure is associated with increased cortisol levels in non-breeding animals compared to the breeding animals (Clarke and Faulkes, 1997). In congruence with this, a higher concentration of CRF receptor binding sites is present in non-breeding naked mole-rats (Beery et al., 2016). In addition, CRF receptor binding sites also differ between solitary and social mole-rat species (Coen et al., 2015), where differences in proliferating cells have been shown. Stress hormones are also expressed in response to running and exercise, where it is associated with an upregulation of AHN (Stranahan et al., 2006). Non-breeding Damaraland mole-rat females show higher levels of locomotor activity than the breeding females (Oosthuizen and Bennett, 2015), therefore increased activity could, at least in part, explain the higher AHN in non-breeding mole-rats.

### Age and longevity

In agreement with their increased life expectancies, mole-rats have long gestation times and subsequent postnatal development is slower than other rodents. Like other animals, mole-rats also show a steep decline in neurogenesis at a relatively young age (Penz et al., 2015). A slow postnatal development may increase the expected window for higher levels of neurogenesis seen in all young animals between birth and puberty (Penz et al., 2015). In mole-rats, puberty is reached around the age of 1 year, when neurogenesis should start to decline exponentially (Bennett and Faulkes, 2000; Penz et al., 2015), however this has not been tested empirically.

### Cognitive activity

As a result of their lightless environment, subterranean animals rely on other mechanisms such as tactile stimuli and memory to navigate their burrow systems (du Toit et al., 2012). Despite the relatively uniform habitat of the sealed tunnel systems, the difference in tunnel length and complexity appears to be sufficient stimulation to induce learning differences in the different species. Damaraland mole-rats with longer and more complex burrow systems, show superior learning abilities compared to the solitary Cape mole-rat (Costanzo et al., 2009; Oosthuizen et al., 2013), Oosthuizen, unpublished data). The enhanced learning abilities of Damaraland mole-rats are associated with more proliferating cells compared to Cape mole-rats (Amrein et al., 2014; Oosthuizen and Amrein, 2016). Although the basal level of neurogenesis differs with social status in highly social species, there is no corresponding difference in learning abilities.

## Conclusions

Neurogenesis studies performed on conventional laboratory rodents by far outnumber those on wild species. Research on laboratory animals is necessary as laboratory models are very useful and convenient tools for the fundamental understanding of the molecular basis and the regulation of neurogenesis, however they have limitations. Laboratory animals live in a completely uniform habitat without natural predators and are bred to minimize variability. To comprehend the functional and adaptive significance of neurogenesis, the environmental, genetic and physiological variation of natural populations is essential. It is important to appreciate that the adaptive value of neurogenesis is species specific. In many instances, natural animal populations show very different and more varied physiological and neurological responses compared to their laboratory counterparts.

This perspective primarily illustrates the diversity in environmental conditions, social structures and longevity in rodent species. Animal models that display a different set of species-specific features may provide insight into the functional and adaptive significance of adult neurogenesis. Mole-rats differ from conventional laboratory animals in a number of important ways, specifically social structure and longevity. Mole-rats (and indeed other natural rodent populations) are not suggested as a replacement for conventional laboratory rodents in neurogenesis research, but rather to complement the existing body of information. Ultimately, understanding the functional and adaptive context of adult neurogenesis will require research on both laboratory animals and natural rodent populations.

## Author contributions

The author confirms being the sole contributor of this work and approved it for publication.

### Conflict of interest statement

The author declares that the research was conducted in the absence of any commercial or financial relationships that could be construed as a potential conflict of interest.
